# NO and ABA Interaction Regulates Tuber Dormancy and Sprouting in Potato

**DOI:** 10.3389/fpls.2020.00311

**Published:** 2020-04-08

**Authors:** Zhike Wang, Rui Ma, Mengshi Zhao, Fangfang Wang, Ning Zhang, Huanjun Si

**Affiliations:** ^1^College of Life Science and Technology, Gansu Agricultural University, Lanzhou, China; ^2^Gansu Provincial Key Laboratory of Aridland Crop Science, Gansu Agricultural University, Lanzhou, China; ^3^College of Agronomy, Gansu Agricultural University, Lanzhou, China; ^4^Dingxi Academy of Agricultural Sciences, Dingxi, China

**Keywords:** nitric oxide, potato, tuber dormancy, sprouting, NO synthase, abscisic acid, nitrate reductase

## Abstract

In plants, nitric oxide synthase (NOS)-like or nitrate reductase (NR) produces nitric oxide (NO), which is involved in releasing seed dormancy. However, its mechanism of effect in potato remains unclear. In this study, spraying 40 μM sodium nitroprusside (SNP), an exogenous NO donor, quickly broke tuber dormancy and efficiently promoted tuber sprouting, whereas 2-(4-carboxyphenyl)-4,4,5,5-tetramethylimidazoline-1-oxyl-3-oxide (c-PTIO), an NO scavenger, repressed the influence of NO on tuber sprouting. Compared with the control (distilled water), SNP treatment led to a rapid increase in NO content after 6 h and a decreased abscisic acid (ABA) content at 12 and 24 h. c-PTIO treatment significantly inhibited increase of NO levels and increased ABA production. In addition, N^*G*^-nitro-L-arginine methyl ester, an NOS inhibitor, clearly inhibited the NOS-like activity, whereas tungstate, an NR inhibitor, inhibited the NR activity. Furthermore, NO promoted the expression of a gene involved in ABA catabolism (*StCYP707A1*, encoding ABA 8′-hydroxylase) and inhibited the expression of a gene involved in ABA biosynthesis (*StNCED1*, encoding 9-*cis*-epoxycarotenoid dioxygenase), thereby decreasing the ABA content, disrupting the balance between ABA and gibberellin acid (GA), and ultimately inducing dormancy release and tuber sprouting. The results demonstrated that NOS-like or NR-generated NO controlled potato tuber dormancy release and sprouting via ABA metabolism and signaling in tuber buds.

## Introduction

The potato (*Solanum tuberosum* L.) is an important food crop and industrial raw material. Plant dormancy and subsequent germination are physiological processes of active adaptation to the environment. Potato tuber dormancy is defined as an inability to sprout under conditions that are favorable for sprouting (Liu et al., 2015). The dormancy and sprouting of potato tubers are very significant for potato cultivation, tuber production, and industrial processing (Aksenova et al., 2013). In potato cultivation, the dormancy degree of tubers as seed potatoes affects the emergence of seedlings in the field, uniformity, and yield (Sonnewald and Sonnewald, 2014), especially in the two season-cropping areas; excessive dormancy will prolong the sprouting and growth of tubers and finally affects the increase of yield. When tubers are used as food or raw materials for processing, a long dormancy period is essential for transportation and storage, and dormancy release results in a large consumption of water and nutrients, decreasing commodity quality and value (Aksenova et al., 2013). Potato tubers exhibit a certain dormancy period after maturation, which ends with the occurrence of buds. The release of dormancy was essential for the sprouting of tubers, which was regulated by both internal and external factors (Koornneef et al., 2002). Tuber dormancy was also regulated by a variety of factors and was dependent on plant hormones (Suttle et al., 2012; Sonnewald and Sonnewald, 2014), genetic factors, tuber variety, storage temperature and conditions, and particular signaling molecules, such as nitric oxide (NO) (Noritake et al., 1996) and reactive oxygen species (ROS) (Peivastegan et al., 2019). In many plants, the balance between abscisic acid (ABA) and gibberellin acid (GA) is a major regulator of the dormancy state, in which GA promotes the progression from breaking to sprouting (Wróbel et al., 2017).

NO is a gaseous free radical that can easily diffuse through biofilms. NO plays a role in numerous plant physiological processes (Begara-Morales et al., 2019), such as transport (Sun et al., 2018), germination (Liu et al., 2010; Arc et al., 2013a), flowering (Khurana et al., 2011), metabolism (Hasanuzzaman et al., 2018), and senescence (Sun, 2018). Moreover, NO is a significant signaling molecule that regulates the plant response to various nonbiological and biological stresses (Begara-Morales et al., 2018), such as stomatal closure (Zhang et al., 2019), heat stress (Parankusam et al., 2017), disease (Srinivas et al., 2014), drought (Wang et al., 2016), and programmed cell death (Ma et al., 2010). Exogenous NO treatment was reported to break dormancy and promote germination in seeds of three warm-seasons C_4_ grasses (Sarath et al., 2006).

In mammals, NO synthase (NOS) is the key enzyme that produces NO (Parankusam et al., 2017). There are numerous potential sources of NO in plants, which are predominantly mediated by NOS and nitrate reductase (NR) (Chamizo-Ampudia et al., 2017). In the presence of NADPH as an electron donor, NOS catalyzes the conversion of L-arginine to L-citrulline and NO (Stuehr et al., 2004), whereas NR reduces nitrates to NO (Gupta et al., 2011). Although the occurrence of NOS in plants has not yet been definitively demonstrated, some studies have indicated that NOS-like activities existed in many plants (Parankusam et al., 2017). These NOS-like activities appeared to be somewhat similar to mammalian NOS (Del Río et al., 2004). Moreover, N^*G*^-nitro-L-arginine methyl ester (L-NAME), an NOS inhibitor, inhibited NO synthesis in plants (Zhang et al., 2007). In addition, tungstate, an NR inhibitor, inhibited NO synthesis in *Arabidopsis* (Kolbert et al., 2010).

Sodium nitroprusside (SNP) is an NO donor that induces the production of NO. Exogenous SNP treatment was reported to break dormancy of barley (Bethke et al., 2004). 2-(4-carboxyphenyl)-4,4,5,5-tetramethylimidazoline-1-oxyl-3-oxide (c-PTIO) is an NO scavenger that blocks the effects of NO donors and prolongs the dormancy in *Arabidopsis* (Liu et al., 2009). Some researchers have demonstrated interaction between NO signal transduction and ABA (Zhang et al., 2007; Arc et al., 2013a). For example, NO was involved in stomatal closure induced by ABA (Zhang et al., 2019). However, the crosstalk between NO and ABA in potato tuber dormancy and sprouting currently remains unclear. Therefore, this study focused on the influence of NO on potato tuber sprouting and investigated the relationship between ABA metabolism and NO signaling in tuber buds and the roles of NOS-like and NR in tuber sprouting.

## Materials and Methods

### Plant Materials

The potato (*S. tuberosum* L.) cultivar “Favorita” was used in the study. The potatoes were planted in Dingxi Academy of Agricultural Sciences, Dingxi City, Gansu Province, China. The harvested tubers were stored at room temperature (25°C ± 1°C) (approximately 90% humidity) in the dark until fully mature. According to report, it takes approximately 1 week for harvested tubers to reach full maturity. When tubers mature completely, cell division is mainly concentrated in the epidermis and periderm cells. The inner cells of the tuber mainly accumulate and develop starch grains. The cell volume typically stops expanding, and few cells divide, whereas starch accumulation is very rich. The degree of vacuolation of cells continues to decrease and is becoming dormant (Teper-Bamnolker et al., 2012). SNP, L-NAME, tungstate, ABA, and c-PTIO were obtained from Beijing Biomarker Technology Company (Beijing, China).

### Tuber Sprouting Test

Healthy tubers with similar size (70–80 mm in diameter) were selected. Dormant tubers were sprayed with distilled water (DW; control) or 10, 20, 40, 80, 160, or 320 μM SNP for 15 min. There were three repetitions per treatment and 50 tubers per repetition. The treated tubers were placed in a box and stored in a cool and well-ventilated place. The sprouting rate was counted every 10 days to determine the optimal SNP concentration. The tuber sprouting rate (%) = (sprouted tuber number/tuber total) × 100. The sprouted tuber means that the first bud of the tuber reaches 2 mm as the sprouting standard (Abbasi et al., 2015). This SNP concentration was then used in the subsequent combination assay, in which dormant tubers were sprayed with either DW (control), 40 μM SNP, 1 mM c-PTIO, or 40 μM SNP + 1 mM c-PTIO for 15 min. To investigate whether NO improves the tolerance to ABA, dormant tubers were sprayed with either DW (control), 100 μM ABA, 100 μM ABA + 40 μM SNP, or 100 μM ABA + 1 mM c-PTIO for 15 min. The bud eye tissues were sampled at 0, 10, 20, 30, 40, 50, and 60 days (Liu et al., 2015).

### Determination of NO and ABA Contents

The NO content was measured using an enzyme-linked immunosorbent assay (ELISA) kit (Wuhan Purity and Biotechnology Co., Ltd., Wuhan, China) according to the manufacturer’s instructions. The ABA content was measured using an ELISA kit (Shanghai Jianglai Biotechnology Co., Ltd., Shanghai, China) according to the manufacturer’s instructions, as described by Zhang et al. (2009).

### Determination of NOS-Like and NR Activities

NOS-like activity was measured using a plant NOS ELISA Kit (Shanghai Xinyu Biotechnology Co., Ltd., Shanghai, China) according to the manufacturer’s instructions, as described by Diao et al. (2017). The NR activity was measured as described by Lu et al. (2014). Approximately 1.0 g of tuber bud eye tissues were ground in a mortar, and 10 mL of 50 mM phosphate buffer (pH 7.8) was added. The homogenate was centrifuged at 12,000 revolutions/min for 20 min. The supernatant was transferred to a new centrifuge tube, and the nitrite production was determined by measuring the absorbance at 540 nm using an ultraviolet-visible spectrophotometer.

### RNA Extraction, Reverse Transcriptase–Polymerase Chain Reaction, and Quantitative Reverse Transcriptase–Polymerase Chain Reaction

Fresh samples of RNA were extracted at various time points. The total RNA was isolated using an Easy Pure Plant RNA Kit (Quanshijin, Beijing, China). The extracted RNA was immediately stored at −80°C. RNA samples with an OD_260_/OD_280_ ratio of between 1.98 and 2.0 were used in subsequent experiments. cDNA synthesis was performed using a TransScript One-Step gDNA Removal and cDNA Synthesis Super MixKit (Quanshijin). The cDNA solution was diluted eightfold with nuclease-free water. TransStart Green qPCR SuperMix (Quanshijin) was used to analyze the expression of key genes involved in potato dormancy release. Quantitative reverse transcriptase–polymerase chain reaction (qRT-PCR) was performed using a Light Cycler 96 real-time PCR system (Roche, Basel, Switzerland) in a total reaction volume of 20 μL containing 10 μL 2 × TransStart Green qPCR SuperMix, 1.0 μL each primer (10 μmol/μL), 2.0 μL cDNA, and 6.0 μL double-distilled water. The reactions were mixed gently and incubated at 95°C for 15 min, followed by 40 cycles of 95°C for 30 s, 62°C for 45 s, and 72°C for 60 s. The qRT-PCR primer sequences were as follows: *StNOS-IP* (F5′-ACTTGTCCTGAAGGGAGGGA-3′; R5′-AGACCACGCAAACCTTGTCA-3′), *StNR* (F5′-AACGC TGAAGCATGGTGGTA-3′; R5′-CACCTCAACCTCGAGTGA CC-3′), *StEF1α* (F5′-CAAGGATGACCCAGCCAAG-3′; R5′-TT CCTTACCTGAACGCCTGT-3′), *StCYP707A1* (F5′-CAGGC TTTCAAGCCCGATTC-3′; R5′-TGAAGAGTGTACCGTGG AGA-3′), *StNCED1* (F5′-ACAGCCGGACACCATTTCTT-3′; R5′-CTAAACCGGCGTTTGCAACT-3′). The potato *StEF1α* gene was used as an internal reference gene. The 2^–ΔΔ*Ct*^ method was used to calculate the relative expression level of target genes (Livak and Schmittgen, 2001). All samples were carried out with three biological replicates and three technical replicates (Kundu et al., 2013).

### Statistical Analysis

The data were analyzed using the IBM SPSS Statistics 20 software package (Amonk, New York). The different treatments were compared using Duncan multiple-range test with a significance level of *p* < 0.05. The charts were prepared using GraphPad Prism 8 (San Diego, United States).

## Results

### Effects of SNP and c-PTIO on Tuber Sprouting

**FIGURE 1:**
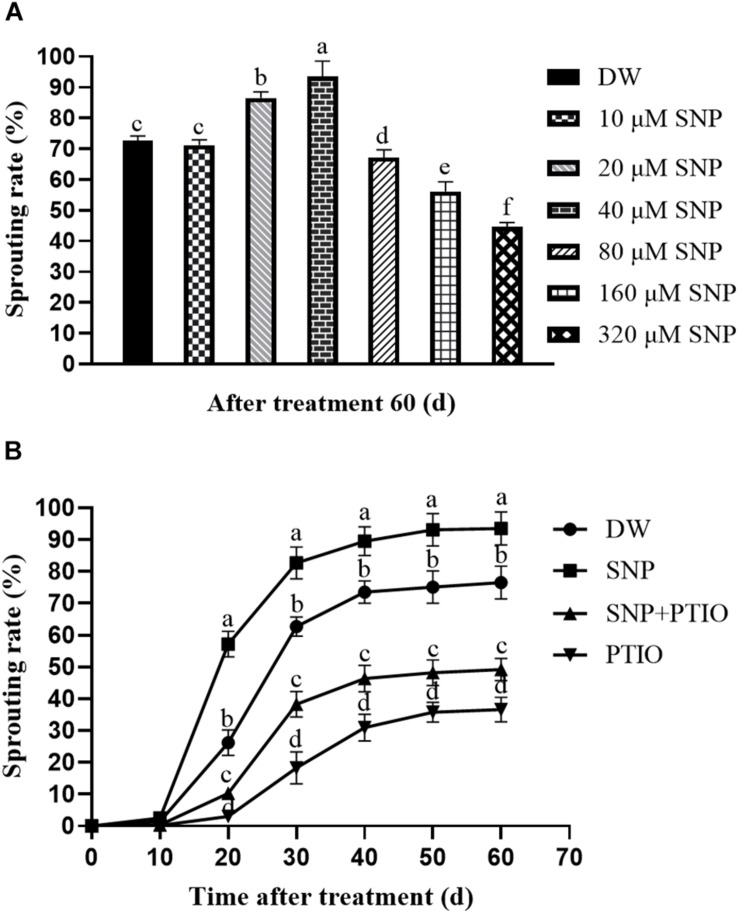
Effects of SNP and c-PTIO on tuber sprouting. **(A)** Effect of different SNP concentrations on tuber dormancy release. Dormant tubers were sprayed with DW (control) or 10, 20, 40, 80, 160, or 320 μM SNP for 15 min. **(B)** Effect of SNP and the NO scavenger c-PTIO on tuber dormancy and sprouting. Dormant tubers were sprayed with DW (control), 40 μM SNP, 1 mM c-PTIO, or 40 μM SNP + 1 mM c-PTIO for 15 min. Data are presented as the mean ± SD for three replicates. When *p* < 0.05, the difference is significant according to Duncan multiple-range test. Values marked with the same letter indicate no significant difference.

Spraying potato tubers with various concentrations of SNP led to sprouting rates of 71.18% (10 μM), 86.55% (20 μM), 93.54% (40 μM), 67.17% (80 μM), 56.12% (160 μM), 44.60% (320 μM), and 72.61% (control) at 60 days after treatment (Figure 1A). In the combination experiment, the tubers sprayed with 40 μM SNP had a sprouting rate of 56.23% at 20 days after treatment, whereas the c-PTIO–treated tubers had a sprouting rate of 3.04%, and the control group sprayed with DW had a sprouting rate of 26.22% (Figure 1B). These results demonstrated that exogenous SNP treatment induced tuber dormancy release and accelerated sprouting. The NO scavenger c-PTIO markedly inhibited tuber sprouting, although combined treatment with both SNP and c-PTIO partially reversed this effect (Figure 1B).

### Effects of SNP and c-PTIO on NO and ABA Contents

**FIGURE 2:**
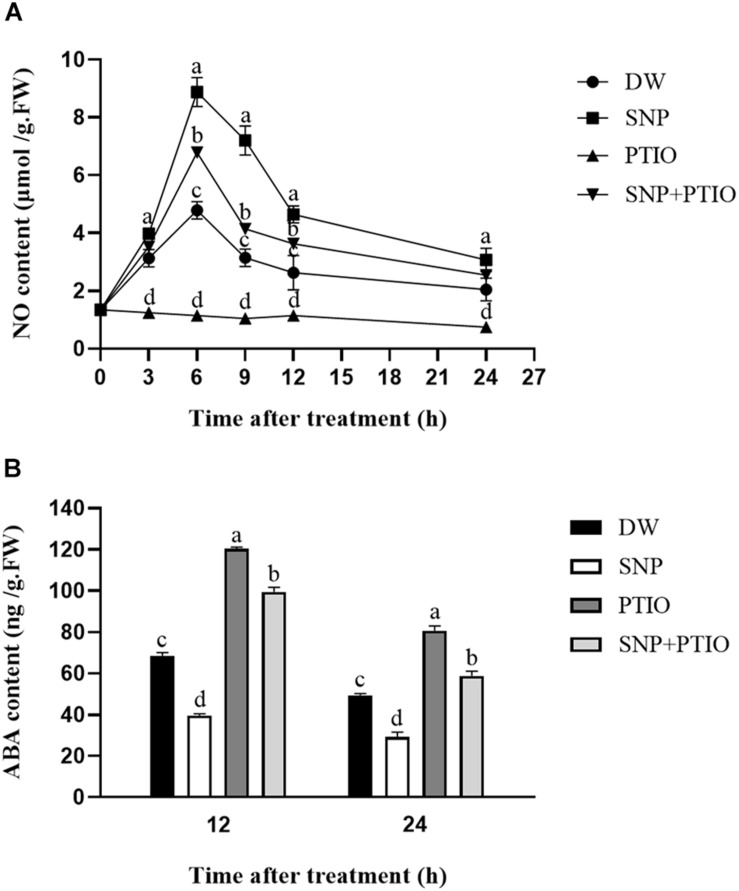
Effects of SNP and c-PTIO on NO **(A)** and ABA **(B)** contents. Dormant tubers were sprayed with DW (control), 40 μM SNP, 1 mM c-PTIO, or 40 μM SNP + 1 mM c-PTIO for 15 min. Data are presented as the mean ± SD for three replicates. When *p* < 0.05, the difference is significant according to Duncan multiple-range test. Values marked with the same letter indicate no significant difference.

The NO and ABA contents were measured after the different treatments. As shown in Figure 2A, compared with the control, SNP treatment led to a rapid increase in the NO content during the first 6 h, which then gradually decreased. Treatment with c-PTIO alone strongly inhibited the increase in NO content, and c-PTIO also partially inhibited the increase in NO content induced by SNP, as shown in Figure 2A. As shown in Figure 2B, compared with the control, SNP treatment decreased the ABA content at 12 and 24 h after treatment, whereas c-PTIO treatment significantly increased the ABA content.

### Influence of Enzyme Inhibitors on Relative Gene Expression

**FIGURE 3:**
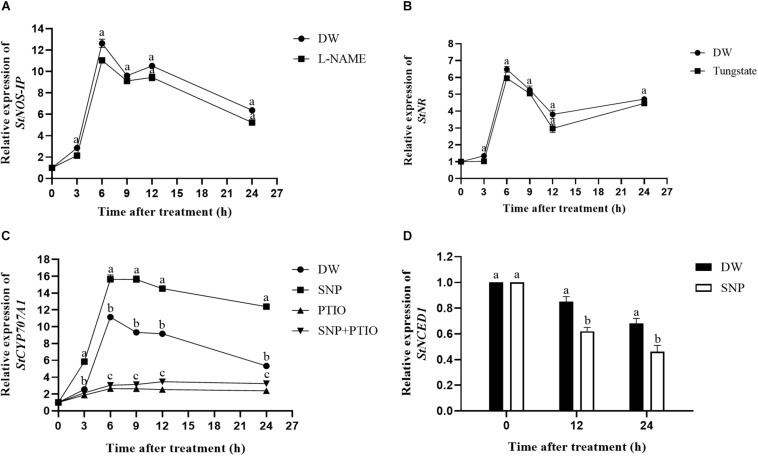
Changes of the relative expression levels of genes (*StNOS-IP*, *StNR*, *StCYP707A1*, and *StNCED1*). **(A)** Effect of L-NAME treatment on the expression of *StNOS-IP* gene. Dormant tubers were sprayed with DW (control) or 1 mM L-NAME for 15 min. **(B)** Effect of tungstate treatment on the expression of *StNR* gene. Dormant tubers were sprayed with DW (control) or 1 mM tungstate for 15 min. **(C)** Effect of SNP treatment on the expression of *StCYP707A1* gene. Dormant tubers were sprayed with DW (control), 40 μM SNP, 1 mM c-PTIO, or 40 μM SNP + 1 mM c-PTIO for 15 min. **(D)** Effect of SNP treatment on the expression of *StNCED1* gene. Dormant tubers were sprayed with DW (control) or 40 μM SNP for 15 min. Data are presented as the mean ± SD for three replicates. When *p* < 0.05, the difference is significant according to Duncan multiple-range test. Values marked with the same letter indicate no significant difference.

The relative expression levels of the *StNOS-IP* (encoding NOS-interacting protein) and *StNR* (encoding NR) genes after enzyme inhibitor treatment were analyzed via qRT-PCR. As shown in Figure 3A, treatment with L-NAME had little effects on the expression level of *StNOS-IP* gene. Similarly, the treatment with tungstate had no obvious influence on the expression level of *StNR* gene (Figure 3B).

To elucidate the role of NO in ABA metabolism in tuber buds, the expression of genes related to ABA metabolism (*StCYP707A1*, encoding ABA 8′-hydroxylase) and ABA biosynthesis (*StNCED1*, encoding 9-*cis*-epoxycarotenoid dioxygenase) was also examined. As shown in Figure 3C, SNP treatment significantly increased the expression of *StCYP707A1* compared with the control, and the highest expression occurred in 6 h after treatment. In contrast, as shown in Figure 3D, SNP treatment significantly decreased the expression of *StNCED1* compared with the control. These results demonstrated that NO promoted the expression of ABA catabolism gene (*StCYP707A1*) and inhibited the expression of ABA biosynthesis gene (*StNCED1*).

### Influence of Enzyme Inhibitors on NOS-Like and NR Activities

**FIGURE 4:**
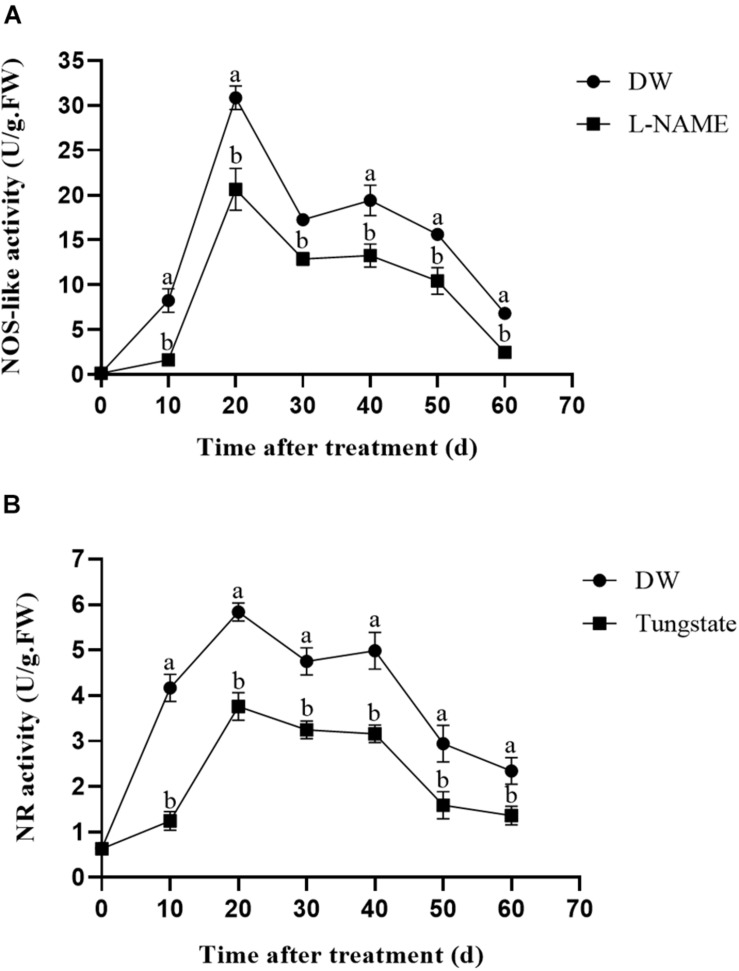
Effect of enzyme inhibitors on NOS-like and NR activities. **(A)** NOS-like activity in potato tubers. Dormant tubers were sprayed with DW (control) or 1 mM L-NAME for 15 min. **(B)** NR activity in potato tubers. Dormant tubers were sprayed with DW (control) or 1 mM tungstate for 15 min. Data are presented as the means ± SD for three replicates. When *p* < 0.05, the difference is significant according to Duncan multiple-range test. Values marked with the same letter indicate no significant difference.

To investigate the effect of the enzyme inhibitors on enzyme activity, the NOS-like and NR activities were measured. As shown in Figure 4A, L-NAME treatment markedly suppressed the NOS-like activity throughout the 60-day observation period compared with the control, and the highest NOS-like activity was observed at 20 days after treatment. A similar trend was observed for the NR activity after tungstate treatment (Figure 4B).

### Influence of NO on ABA Tolerance of Potato During Sprouting

**FIGURE 5:**
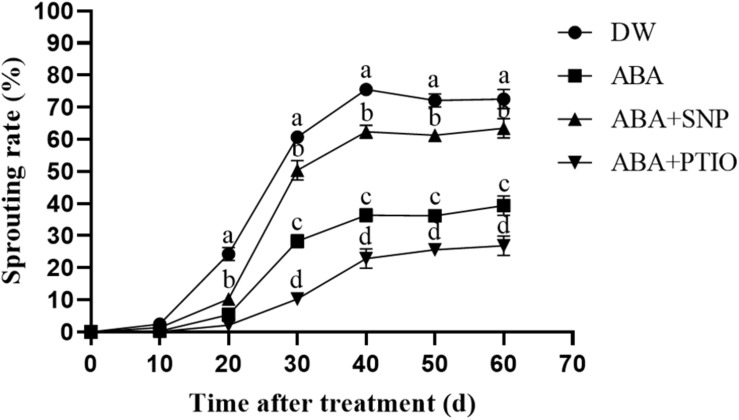
Effect of NO on ABA tolerance of potato during sprouting. Dormant tubers were sprayed with DW (control), 100 μM ABA, 100 μM ABA + 40 μM SNP, or 100 μM ABA + 1 mM c-PTIO for 15 min. Data are presented as the mean ± SD for three replicates. When *p* < 0.05, the difference is significant according to Duncan multiple-range test. Values marked with the same letter indicate no significant difference.

It is well known that ABA effectively inhibits the sprouting of tubers. As shown in Figure 5, the treatment of dormant tubers with 100 μM ABA significantly inhibited sprouting compared with the control. However, upon the addition of 40 μM SNP to ABA, the sprouting rate of the tubers was increased, whereas the addition of 1 mM c-PTIO to ABA caused the sprouting rate to further decrease. These results demonstrated that NO increased the ABA tolerance of potato during sprouting.

### Influence of ABA Treatment on NO Content

**FIGURE 6:**
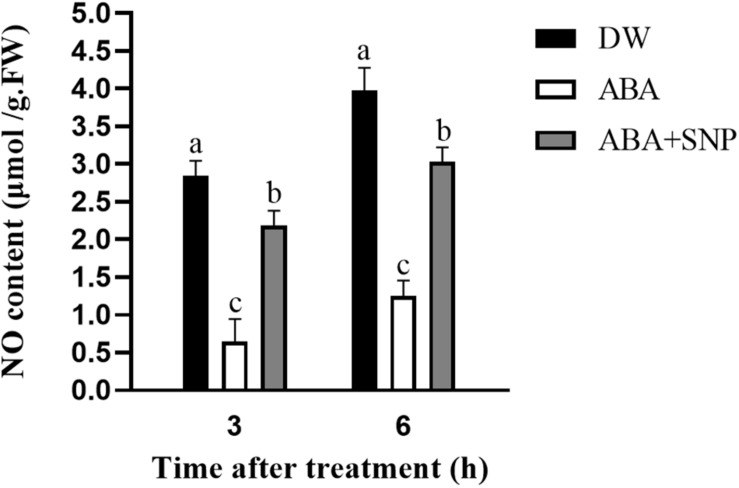
Effect of ABA treatment on NO content. Dormant tubers were sprayed with DW (control), 100 μM ABA, or 100 μM ABA + 40 μM SNP for 15 min. Data are presented as the mean ± SD for three replicates. When *p* < 0.05, the difference is significant according to Duncan multiple-range test. Values marked with the same letter indicate no significant difference.

To examine the effect of ABA on NO, the NO content was measured following ABA treatment. As shown in Figure 6, compared with the control, the NO content was decreased at 3 and 6 h after ABA treatment. The addition of SNP to ABA partially mitigated this decrease at both time points. These results demonstrated that ABA treatment decreased the NO content in potato during sprouting.

### Influence of ABA on Relative Gene Expression

**FIGURE 7:**
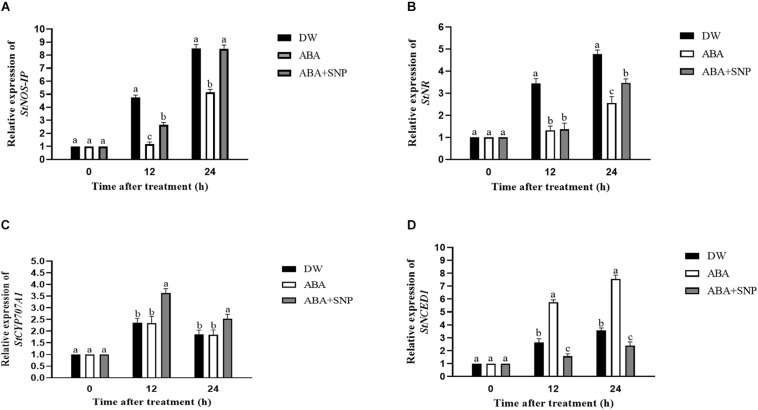
Effect of ABA treatment on the relative expression of genes *StNOS-IP* **(A)**, *StNR* **(B)**, *StCYP707A1* **(C)**, and *StNCED1* **(D)**. Dormant tubers were sprayed with DW (control), 100 μM ABA, or 100 μM ABA + 40 μM SNP for 15 min. Data are presented as the mean ± SD for three replicates. When *p* < 0.05, the difference is significant according to Duncan multiple-range test. Values marked with the same letter indicate no significant difference.

The effects of ABA on the relative expression of the *StNOS-IP*, *StNR*, *StCYP707A1*, and *StNCED1* genes were analyzed via qRT-PCR. As shown in Figures 7A,B, ABA treatment decreased the relative expression of the *StNOS-IP* and *StNR* genes after 12 h compared with the control, whereas ABA + SNP treatment partially mitigated this decrease for both genes. As shown in Figures 7C,D, ABA treatment had little effect on the expression level of the *StCYP707A1* gene, although it increased the relative expression of *StNCED1* gene after 12 h of treatment compared with the control.

### Influence of ABA on NOS-Like and NR Activities

**FIGURE 8:**
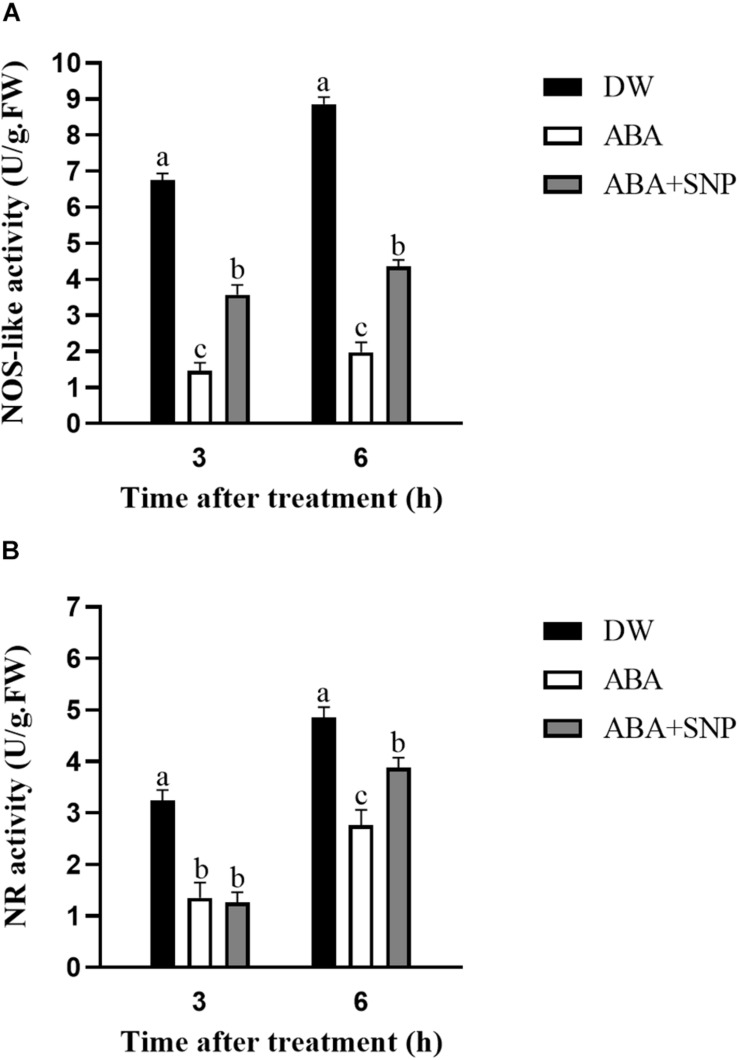
Effect of ABA treatment on NOS-like **(A)** and NR **(B)** activities. Dormant tubers were sprayed with DW (control), 100 μM ABA, or 100 μM ABA + 40 μM SNP for 15 min. Data are presented as the mean ± SD for three replicates. When *p* < 0.05, the difference is significant according to Duncan multiple-range test. Values marked with the same letter indicate no significant difference.

To investigate the effect of ABA on NO, the NOS-like and NR activities were measured following ABA treatment. As shown in Figure 8, the NOS-like and NR activities decreased at 3 and 6 h after ABA treatment compared with the control. The addition of SNP to ABA partially reversed this inhibitory effect for both enzymes. These results showed that ABA treatment inhibited the activities of NOS-like and NR in potato during sprouting.

## Discussion

NO has been demonstrated to play a critical role in dormancy breaking or germination in several plants, including wheat (Bethke et al., 2006a; Jacobsen et al., 2013), barley (Bethke et al., 2004), warm-season C_4_ grass (Sarath et al., 2006), and lettuce (Beligni and Lamattina, 2000). Liu et al. (2009) reported that the NO donor SNP broke seed dormancy, whereas the NO scavenger c-PTIO blocked the effects of NO donors and extended dormancy in *Arabidopsis*. In this study, the exogenous 40 μM SNP treatment quickly broke the dormancy of tubers and promoted their rapid sprouting (Figure 1A), whereas the NO scavenger c-PTIO markedly delayed tuber sprouting (Figure 1B). However, combined treatment with SNP and c-PTIO partially reversed the effect of c-PTIO (Figure 1B). In plant research, the most common NO donors are SNP, S-nitrosopenicillamine (SNAP), S-nitrosoglutathione (GSNO), and diethylamine NONOate (DETA/NO). Different donors have different mechanisms of NO release. For example, SNP releases the nitrosonium cation (NO^+^), whereas SNAP and GSNO typically release the NO radical (^.^NO). In aqueous solution, DETA/NO and SNAP produce instantaneous NO bursts lasting seconds to minutes, whereas the NO release effect of SNP is more prolonged (Planchet and Kaiser, 2006; Mur et al., 2013). SNP is one of the most extensively studied NO donors and can be used for the persistent production of NO (Mur et al., 2013). However, there are some shortcomings with the application of SNP. For example, the release of NO from SNP is accompanied by the production of the toxic gas hydrogen cyanide (Bethke et al., 2006b). According to the report of Oracz et al. (2009), SNP produced cyanide that was likely to induce ROS generation, for instance, hydrogen peroxide (H_2_O_2_). Because of time constraints, H_2_O_2_ content could not be measured in time in this study. In *Arabidopsis* and barley seed, c-PTIO enhanced their dormancy (Bethke et al., 2004). In short, our findings are consistent with those reports for other species.

The recent plans to deregister Chlorpropham (CIPC) in European refer to germination inhibitors and herbicide CIPC have been banned use by the Council of Europe since January 1, 2020. The European Commission has published its 2019/989 implementing regulations on the nonextension of the approved active substance CIPC. According to this ordinance, the CIPC authorization has not been renewed. Member states should countermand their authorization for plant protection products involving CIPC as an active substance by January 8, 2020. CIPC is a highly efficient potato bud inhibitor (Frazier and Olsen, 2015). In the past, some export regulations required that potatoes must be treated with CIPC or other bud inhibitors as a plant disease prevention measure. However, CIPC is slightly toxic and remains in the potato tubers after application, which could cause harm to people and the environment (Abbasi et al., 2015). In an effort to effectively inhibit sprouting and reduce the occurrence of diseases during storage, tubers are typically stored at low temperature (Ou et al., 2014). Under the conditions of low-temperature storage, the content of reducing sugars in the tubers rapidly increases (Malone et al., 2006). During high-temperature processing, these reducing sugars react with free amino acids in tubers via the Maillard reaction (Claassen et al., 1993), which severely affects the color of fried tuber strips and flakes. Because the content of reducing sugars in potato chips is negatively correlated with the color of the chips, a higher reducing sugar content leads to decreased quality. The change in reducing sugar content in potato tubers is closely related to the temperature. When the storage temperature of tubers was lower than 10°C, the reducing sugar content increased, and the sucrose content also increased significantly. At 10°C, although the reducing sugar content increased slightly, the sucrose content remained essentially unchanged.

Detailed morphological analysis revealed that bud growth was not observed when tubers were dormant after harvest (Van Ittersum et al., 1992). At the molecular level, the meristem of the dormant tuber bud is rarely divided. When the buds begin to grow after the end of the dormancy (Leshem and Clowes, 1972), the cells in the middle stage of division are also increasing. Although mitosis is in a resting state, the resting meristem still possesses metabolic activity. Numerous studies have demonstrated that dormant meristems capture and incorporate precursors of RNA and proteins.

The NO and ABA contents were measured after the various treatments. Compared with the control, SNP treatment caused a rapid increase in the NO content at 6 h, which then gradually decreased, whereas c-PTIO treatment strongly inhibited the increase in NO content (Figure 2A). The addition of c-PTIO also partially mitigated the increase in the NO content caused by SNP (Figure 2B). Liu et al. (2009) reported that the fluorescence intensity was increased at 3 h before inhibition in *Arabidopsis* seeds and decreased after 6 h, while the fluorescence intensity was decreased after c-PTIO treatment. However, owing to technical and experimental constraints, we failed to detect NO using the fluorescent dye DAF-FMDA. The ABA content decreased at 12 and 24 h after treatment with SNP, whereas c-PTIO treatment significantly increased the ABA content (Figure 2B).

NOS-like and NR activities are two primary enzymatic sources of NO in plants (Parankusam et al., 2017). An increasing number of studies have demonstrated the presence of NOS-like activity in plants and its similarity to the mammalian enzyme (Del Río et al., 2004). Moreover, L-NAME, an NOS inhibitor, inhibits NO production in plants (Zhang et al., 2007), and tungstate, an NR inhibitor, was reported to inhibit NO production in *Arabidopsis* (Kolbert et al., 2010). To assess the effect of these enzyme inhibitors on enzymatic activity, the NOS-like and NR activities were measured. Compared with the control, L-NAME treatment markedly suppressed the NOS-like activity throughout the duration of the experiment, and the NOS-like activity reached its highest level at 20 days after treatment (Figure 4A). The same trend was observed for the NR activity following tungstate treatment (Figure 4B). Furthermore, the relative expression levels of the *StNOS-IP* and *StNR* genes were analyzed via qRT-PCR after enzyme inhibitor treatment. The results revealed that L-NAME treatment had no effect on the expression of the *StNOS-IP* gene (Figure 3A), and tungstate treatment had no effect on the expression of the *StNR* gene (Figure 3B). The *StNOS-IP* gene encoding NOS-interacting protein was obtained from the National Center for Biotechnology Information (NCBI) database^1^ and was blasted in the Potato Genome Sequencing Consortium (PGSC) database^2^. The results revealed that this gene was predicted in the NCBI database (LOC102602935), but was a conserved gene of unknown function in the PGSC database (PGSC0003DMT400057534). We downloaded the sequence of this gene from the NCBI database and designed qRT-PCR primers. The gene possessed a total length of 1234 bp, a coding sequence length of 915 bp, and a translated protein length of 304 amino acids and contained two exons. However, the expression of the *StNOS-IP* gene had effect on the activity of NOS-like. How this gene influences the enzyme activity is unclear and will need further study.

It has been suggested that NO is involved in mediating ABA catabolism. The *CYP707A* families of genes, which encode ABA 8′-hydroxylases, are responsible for regulating ABA catabolism (Sanz et al., 2015). The mechanism underlying the effect of NO on potato tuber dormancy remains unclear at present. To elucidate the role of NO in ABA metabolism in tuber buds, the expression of genes involved in ABA catabolism (*StCYP707A1*) and ABA biosynthesis (*StNCED1*) was also examined. The expression of *StCYP707A1* after SNP treatment was significantly higher than that in the control, and the highest expression occurred at 6 h after treatment (Figure 3C). In contrast, the expression of *StNCED1*after SNP treatment was significantly lower than that in the control (Figure 3D). These results demonstrated that NO promoted the expression of *StCYP707A1* and inhibited the expression of *StNCED1*.

It is well known that ABA inhibits seed germination. At present, it is generally believed that ABA is a positive regulator of dormancy induction, which is involved in the maintenance of dormancy and the decrease of endogenous ABA content during the release of dormancy (Campbell et al., 2008; Suttle et al., 2012). The use of the ABA inhibitors and the discovery of a synthetic mutant of ABA confirmed that ABA inhibits the release of dormancy, although this inhibitory effect can be reversed by GA. The crosstalk between ABA and NO regulating dormancy and germination in seed has been reported. Used exogenous SNP, an NO donor, promote NO production, which inhibits ABA synthesis and promotes ABA catabolism. According to the report of ABA crosstalk with ethylene and NO in seed dormancy and germination (Arc et al., 2013b), the signal transduction between ABA and NO was realized by PYR/PYL/RCAR receptor (Staszak et al., 2017). When ABA does not combine with the receptor, 2C protein phosphatase (PP2C) dephosphorylates sucrose nonfermenting 1–related protein kinase 2 (SnRK2) (Manohar et al., 2017). When ABA combining with the receptor, induces the formation of a protein complex with PP2C (Staszak et al., 2017) and releases inhibition of SnRK2 activity, which can phosphorylate downstream targets, containing ABA insensitive 5 (ABI5)-related transcription factors (Lim et al., 2015). Interactions between ABI3 and ABI5 mediate transcriptional regulation of ABA-responsive genes (Arc et al., 2013b). In our study, we suppose that treatment with exogenous SNP, an NO donor, activates NOS-like or NR activity, thereby promoting NO production. Subsequently, NO promotes the catabolism of ABA and inhibits its biosynthesis. Therefore, the ABA content is decreased, disrupting the balance between ABA and GA. Finally, dormancy release and tuber sprouting occur. However, the specific molecular mechanism of NO and ABA interaction regulating the dormancy and sprouting of potato tubers remains unclear and will need further study.

The cotreatment of seeds with 200 μM SNP and 10 μM ABA was reported to promote the embryo root growth after 4 days, compared with treatment with ABA alone (Sarath et al., 2006). In addition, NO effectively improved the tolerance of *Arabidopsis* seeds to ABA (Liu et al., 2009). In our study, treatment of dormant tubers with 100 μM ABA significantly inhibited tuber sprouting compared with the control (Figure 5). However, upon adding 40 μM SNP to ABA, the sprouting rate of the tubers increased, and upon adding 1 mM c-PTIO to ABA, the sprouting rate of the tubers further decreased. These results demonstrated that NO improved the ABA tolerance of potato during sprouting.

To study the influence of ABA on NO, the NO content was measured following ABA treatment. As shown in Figure 6, compared with the control, the NO content decreased at 3 and 6 h after ABA treatment. The addition of SNP to ABA partially reversed this decrease at both time points. These results indicated that ABA treatment decreased the NO content in potato during sprouting. The NOS-like and NR activities were also measured following ABA treatment. Both activities decreased at 3 and 6 h after ABA treatment compared with the control, and the addition of SNP to ABA reversed the inhibitory effect of ABA on the activities of these two enzymes (Figure 8). These results showed that ABA treatment inhibited both the NOS-like and NR activities in potato tuber during sprouting.

The effects of ABA on the relative expression levels of the *StNOS-IP*, *StNR*, *StCYP707A1*, and *StNCED1* genes were analyzed by qRT-PCR. ABA decreased the relative expression of the *StNOS-IP* and *StNR* genes after 12 h of treatment (Figures 7A,B) compared with the control, whereas ABA + SNP treatment partially mitigated this decrease for both genes. ABA treatment had little influence on the expression level of the *StCYP707A1* gene, although it increased that of *StNCED1* gene after 12 h of treatment compared with the control (Figures 7C,D).

**FIGURE 9:**
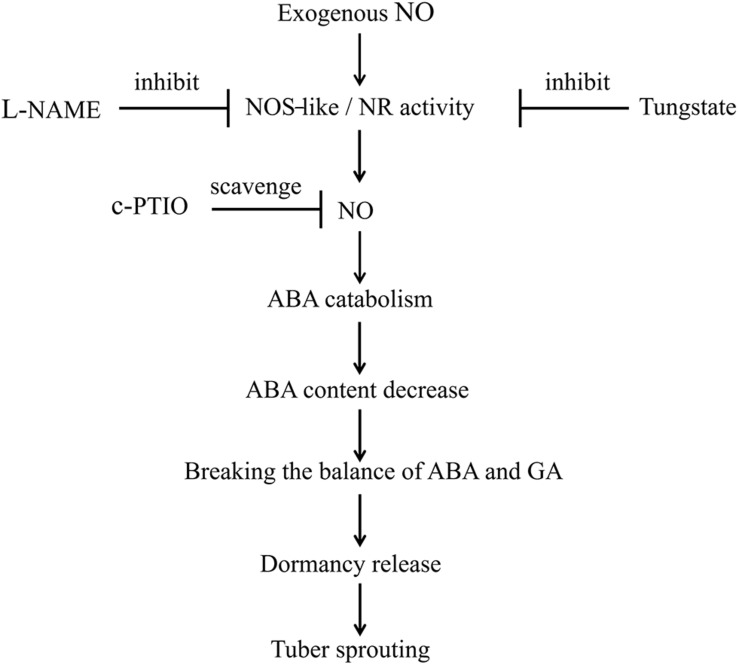
Model depicting how NO, ABA, NOS-like, and NR control tuber dormancy and sprouting. Exogenous SNP spraying activates NOS-like or NR activity, which promotes NO production. Subsequently, NO promotes the catabolism of ABA and inhibits its biosynthesis. The resulting decreased ABA content disrupts the balance between ABA and GA, ultimately leading to dormancy release and tuber sprouting. In addition, L-NAME inhibits NOS-like activity, tungstate inhibits NR activity, and c-PTIO scavenges NO. Overall, the NOS-like or NR-generated NO controls potato tuber dormancy release and sprouting via ABA metabolism and signaling in tuber buds.

In summary, treatment with exogenous SNP, an NO donor, activates NOS-like or NR activity, thereby promoting NO production. Subsequently, NO promotes the catabolism of ABA and inhibits its biosynthesis. Therefore, the ABA content is decreased, disrupting the balance between ABA and GA. Finally, dormancy release and tuber sprouting occur. In addition, L-NAME and tungstate inhibit the activities of NOS-like and NR, respectively, and c-PTIO scavenges NO. Ultimately, the NOS-like or NR-generated NO controls potato tuber dormancy release and sprouting via ABA metabolism and signaling in tuber buds (Figure 9). However, the interactions between NO, GA, and ABA during the tuber dormancy release process are highly complex, and the underlying mechanisms require further study.

## Data Availability Statement

The datasets generated for this study are available on request to the corresponding author.

## Author Contributions

NZ and HS conceived and designed the experiments. ZW, RM, MZ, and FW performed the laboratory experiments. ZW, MZ, and FW performed the data analysis and interpretation. ZW, NZ, and HS wrote the manuscript.

## Conflict of Interest

The authors declare that the research was conducted in the absence of any commercial or financial relationships that could be construed as a potential conflict of interest.

## Footnotes

1 https://www.ncbi.nlm.nih.gov

2 http://solanaceae.plantbiology.msu.edu/pgsc_download.shtml
